# Unravelling the unique essential genes of Streptococcus canis through transposon-directed insertion-site sequencing

**DOI:** 10.1099/mgen.0.001701

**Published:** 2026-05-12

**Authors:** Etienne Aubry, Miriam M.D. Katsburg, Dennis Hanke, Inga Eichhorn, Silver A. Wolf, Anna Kopenhagen, Simone Bergmann, Andrew Waller, Julian Parkhill, Marcus Fulde

**Affiliations:** 1 Centre of Infection Medicine, Institute of Microbiology and Epizootics, Freie Universität Berlin, Berlin, Germany; 2 Genome Competence Centre (MF1), Robert Koch Institute (RKI), Berlin, Germany; 3 Institute of Microbiology, Technische Universität Braunschweig, Braunschweig, Germany; 4 Intervacc AB, Hagersten, Sweden; 5 Department of Veterinary Medicine, University of Cambridge, Cambridge, UK; 6 Veterinary Centre for Resistance Research (TZR), Freie Universität Berlin, Berlin, Germany

**Keywords:** essentialome, *ldh*, *pta*, *Streptococcus*, transposon-directed insertion-site sequencing (TraDIS)

## Abstract

*Streptococcus canis* represents a major canine pathogen, accounting for 22.4% of streptococcal infections in dogs. However, despite its prevalence in veterinary medicine, the mechanisms underlying *S. canis* pathogenesis and survival remain poorly understood. Identifying targeted treatments against *S. canis* could help to reduce dysbiosis-related complications and minimize the selection of resistant neighbouring bacteria. In this study, we employed transposon-directed insertion-site sequencing for the first time to generate saturated mutant libraries of *S. canis*. By comparing three distinct strains, we defined the shared essential genome of this pathogen. We found that 90.4% of its essential genes are also present in the essential genomes of other related pyogenic streptococcal species, including *Streptococcus pyogenes*, *Streptococcus agalactiae* and *Streptococcus equi* subsp. *equi*, demonstrating the translational relevance of *S. canis* research to broader streptococcal biology. Notably, we identified two genes uniquely essential to *S. canis* at the terminal steps of glycolysis: *ldh*, which governs lactate metabolism, and *pta*, which catalyses the conversion of acetyl-CoA to acetyl phosphate in acetate metabolism. We propose that targeting these pathways may offer a novel, species-specific therapeutic strategy for treating *S. canis* infections.

**Impact Statement**

Transposon sequencing enables the creation of dense mutational libraries to characterize the impact of various mutations across the genome of a given bacterial organism. Here, we have conducted transposon-directed insertion-site sequencing (TraDIS) on the veterinary pathogen, *Streptococcus canis*, which is known to primarily affect companion animals such as dogs and cats. For the first time, we have produced the essential genome of *S. canis* using TraDIS and compared it to other closely related pathogens within the *Streptococcus* pyogenic species group. We highlight that the essential genome of *S. canis* is comparable to that of *Streptococcus pyogenes*, *Streptococcus agalactiae* and *Streptococcus equi* subsp. *equi*. This has strong implications for the translatability of research between these different pathogens. Furthermore, we have identified a set of unique essential genes found within the three *S. canis* isolates we have used in this study. These genes could one day serve as targets for the treatment of *S. canis* infections. Here, we provide a major step towards understanding the overall mechanisms behind *S. canis* survival and how to encourage intervention against infection without causing dysbiosis.

## Data Summary

TraDIS reads for *Streptococcus canis* were submitted to the NCBI Sequence Read Archive (SRA) under Bioproject number PRJNA1312318 (SRS26336389, SRS26336388, SRS26336387 for AGF1037, AGF1032 and AGF957, respectively).

TraDIS reads for *Streptococcus equi* were taken from SRP102055.

TraDIS reads for *Streptococcus agalactiae* were taken from PRJNA674399.

TraDIS reads for *Streptococcus pyogenes* were taken from PRJNA494385.

The phylogenetic tree was generated with sequence reads from Bioproject number PRJNA1332248 and public repositories (accession numbers available in Table S1).

## Introduction

*Streptococcus canis* is a Lancefield group C and G *β*-haemolytic pathogen [1,3]. It is a member of the residential microbiome of dogs and cats and can cause opportunistic infections such as keratitis and otitis externa [4]. In rare cases, it has also been reported to cause more severe systemic infections such as streptococcal toxic shock-like syndrome, necrotizing fasciitis and infective endocarditis [5,7]. *S. canis* represents 22.4% of streptococcal canine infections, establishing it as a major pathogen within dogs [8]. However, several studies have shown that *S. canis* can infect a diverse range of hosts, including cattle, humans, seals, rats and mink [2,9,13]. This makes it similar in host range to its pyogenic relative, *Streptococcus agalactiae*, which is capable of infecting humans, cattle and fish, while remaining distinct from the human-restricted pathogen, *Streptococcus pyogenes*, and the equine-restricted pathogen, *Streptococcus equi* subsp. *equi* [14,16].

Whole-genome sequencing has seen a recent rise in accessibility, and with it an emergence of novel methods which are able to better characterize the genetic variations of today’s pathogens [17]. In particular, new advances in sequencing technologies have allowed precise strain classifications which can be used to associate virulence factors, pathogenic markers and antimicrobial resistance genes with emerging lineages which have been notably applied to *S. pyogenes*, *S. equi*, *S. agalactiae* and *S. canis* [14,18,20]. However, to further enable such associations, a baseline knowledge of the pathogenesis of these specific micro-organisms is essential. This is not the case for *S. canis*, as the universally present M protein (SCM) represents one of its only thoroughly characterized virulence factors, with previous studies observing its capacity to bind (mini)-plasminogen and IgG for SCM-I and fibrinogen for SCM-II [21,24]. Recently, IdeC, an IgG-specific protease, has been characterized as another important virulence factor for *S. canis* capable of cleaving IgG in dogs, cats and humans, but still remains only the second pathogenesis marker of *S. canis* that has majorly been studied [25]. To compensate for this, a new method that allows a global approach for evaluating the fitness of new pathogens within the context of infection and laboratory growth was designed: transposon-insertion sequencing (TIS) [26]. TIS has been increasingly applied since the publication of four variations of the method in 2009: transposon sequencing (Tn-Seq), insertion sequencing (INSeq), high-throughput insertion tracking by deep sequencing (HITS) and transposon-directed insertion-site sequencing (TraDIS) [27,30]. Whereas Tn-Seq and INSeq use restriction enzymes to generate libraries with DNA reads of uniform length, TraDIS and HITS use a random DNA shearing method to generate accurate mapping of the transposon in the genome of the targeted pathogen [31].

**Fig. 1.**
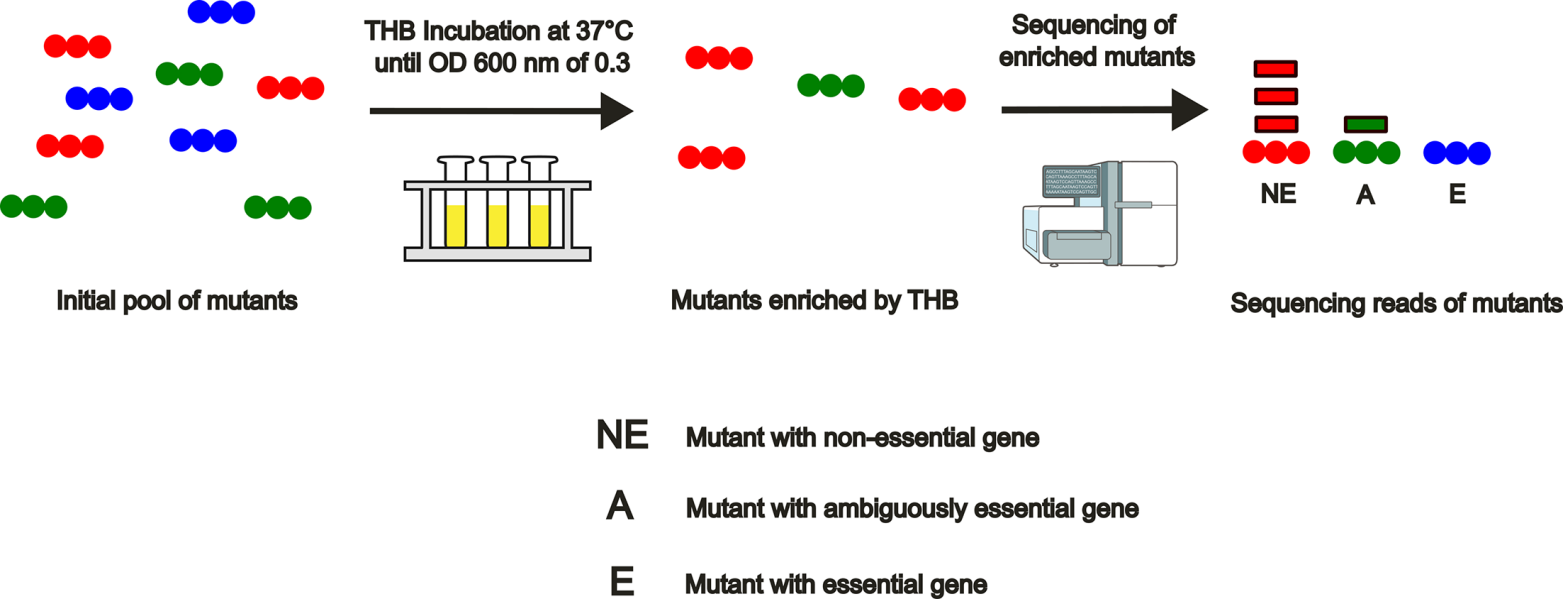
Graphical representation of the TraDIS method from the initial library mutant pool to the calling of gene essentiality.

Following the establishment of TIS came the development of efficient ways to study the essential genome. A gene is considered essential if it is needed for the survival or reproductive success of an organism. The essential genome is the set of genes that are designated as essential within a given organism [32]. The TraDIS method is used to generate a random pool of mutants, which will be grown in media to enrich the metabolically advantaged strains (Fig. 1). The enriched mutants are then sequenced and their reads counted to assess gene essentiality. Mutants that have a number of reads passed a certain threshold will be classified as having non-essential genes. Mutants that are significantly below this threshold will be counted as having essential genes. Any mutants that are neither clearly essential nor non-essential will be classified as ambiguous (Fig. 1). Earlier essentiality studies have been conducted on *S. pyogenes* showing that most vital genes could be attributed to major cellular processes such as peptidoglycan biosynthesis and glycolysis [33]. The essential genome of *S. agalactiae* was also characterized, showing 93% concordance with that of *S. pyogenes* [34]. A study centred on the comparison of the essential genomes of *S. pyogenes*, *S. agalactiae* and *S. equi* subsp. *equi* revealed significant overlap of genes across all three pathogens, indicating a major opportunity for translational research between them [35]. Interestingly, this same study also identified essential genes unique to *S. equi* subsp. *equi*, such as *eqbA* – the regulator of the equibactin locus. This demonstrates how Tn-Seq can elucidate vital mechanisms specific to a given pathogen. We hypothesize that the genome of *S. canis* also displays significant overlap in its essential genome when compared to other pyogenic streptococci, but that it might have a subset of unique genes that may be used as specific targets for potential treatment.

This study therefore aims to produce the first shared essential genome of *S. canis* compared to *S. pyogenes*, *S. agalactiae* and *S. equi* subsp. *equi* using TraDIS. For this, Bayesian analysis of the population structure (BAPS) was applied to 598 public *S. canis* isolates to cluster the population based on SNP differences in the whole genome and identify the most prominent lineages. Two isolates from the recently emerged and pathogenic BAPS-6 canine cluster and one isolate from the multi-host canine, feline and bovine BAPS-5 cluster were selected for the succeeding analysis. This enabled the identification of overlaps in the essential genome of *S. canis* compared with other streptococci and the identification of genes which are uniquely essential to *S. canis*.

## Methods

### Generation of transposon libraries

*S. canis* strains were first cultivated on Columbia blood agar plates with 5% sheep’s blood at 37 °C overnight. Cells were transformed with the pGh9:ISS1 plasmid (NCBI: txid481005) by electroporation as previously described [21,36, 37]. Briefly, competent cells were generated via growth in brain heart infusion (BHI) media overnight without shaking at 37 °C and inoculation of 100 µl of the culture into a new BHI tube until an OD of 0.3 at 600 nm was achieved. The resulting culture is then centrifuged three times at 14,000 r.p.m. while being resuspended in Millipore water to eliminate any residual BHI media and finally resuspended in 10% glycerol completed with Millipore water to create 100 µl aliquots, which were stored at −80 °C until transformation. Competent cells were defrosted on ice and 1 µg of pGh9:ISS1 was added to each aliquot for a 5 min incubation. Competent cells were then electroporated with 1 mm cuvettes via a pulse of 2.5 kV with a resistance of 200 Ω. BHI media was added to recover the cells for 3 h at 28 °C without shaking. The transformants were then plated on BHI agar with 2 µg ml^−1^ of erythromycin at 28 °C for 3 days and resistant colonies were picked and grown in BHI with 2 µg ml^−1^ of erythromycin overnight. Overnight cultures were heat shocked at 40 °C for 3 h. Transposants were selected by overnight growth at 37 °C in a humidified atmosphere containing 5% CO2 on 50 BHI agar plates supplemented with 2 µg ml^−1^ of erythromycin. Pools of random transposon mutants (transposon libraries) were harvested from the plates by washing with BHI containing 25% glycerol and the bacterial suspension was stored at −20 °C. The transposon libraries were then grown at 37 °C to an OD 600 nm of 0.3 in Todd Hewitt Broth (THB) with 2 µg ml^−1^ of erythromycin. Briefly, 2.5 ml of the culture was centrifuged at 10,000 ***g*** for 5 min and the bacterial pellet was stored at −80 °C for sequencing.

### Sequence assembly and phylogenetic tree

Samples were taken from a previous *S. canis* study where AGF1032, AGF1037 and AGF957 were selected for the TraDIS analysis [20]. FastQ files were quality assessed with *FastQC* v0.12.1 (https://www.bioinformatics.babraham.ac.uk/projects/fastqc/). The raw reads were then put through the *Bactmap* v. 1.0.0 pipeline for quality and adapter trimming via the integrated *fastp* v0.23.4 programme [38]. The entire dataset was then mapped to the HL_77_2 *S. canis* reference genome (NCBI RefSeq assembly GCF_010993845.2). *Gubbins* v3.3 was run independently on the mapped assemblies to remove loci associated with recombination events under default settings [39]. Maximum likelihood phylogenetic trees were constructed from reference-based alignments produced after masking recombinant regions. *IQtree* v2.0.3 was run on these alignments independent of the pipelines while accounting for constant sites using the best fit model (MODEL) and 1,000 bootstrap replicates [40,43].

### DNA preparation and sequencing by TraDIS

DNA was extracted from the mutant libraries after overnight growth at 37 °C in THB supplemented with 2 µg ml^−1^ of erythromycin (*n*=1 for each strain). The QIAamp DNA Mini Kit (QIAGEN, Venlo, Netherlands) was used following the standard Gram-positive protocol. DNA was quantified using the Qubit dsDNA HS assay kit. Briefly, 500 ng of DNA per replicate was fragmented enzymatically using NEBNext Ultra II FS DNA library prep kit for Illumina (New England Biolabs #E7805, E6177) to produce fragments of 600 bp. A Y-adaptor was generated in-house using Illumina multiplexing adaptor sequences (Oligonucleotide sequences ©2007–2012 Illumina, Inc. All rights reserved) according to previous methods [31]. This was ligated to the fragmented DNA using the aforementioned NEBNext Ultra II DNA library prep kit according to the manufacturer’s instructions. Fragments were purified using AMPure XP beads (Agencourt, Beckman Coulter) with a bead to DNA ratio of 1:1, according to manufacturer’s instructions.

Adaptor ligated DNA was then incubated with the restriction enzyme SmaI for 2 h at 25 °C. A NEB PCR purification kit (New England Biolabs) was used to remove the restriction enzyme according to manufacturer’s instructions. The amount of DNA recovered was quantified using the Qubit dsDNA HS assay kit. Briefly, 100 ng of library DNA was PCR amplified for 20 cycles according to the NEBNext Ultra II DNA library prep kit protocol. Amplification utilized the specific ISS1 primer and indexing PCR primers from previous publications (Table S9, available in the online Supplementary Material) [35]. AMPure XP beads with a bead to DNA ratio of 0.8:1 were used to remove small PCR products, non-ligated adaptors and primer dimers. The concentrations of the libraries were calculated using the Qubit dsDNA HS assay kit, with average fragment sizes estimated from gel electrophoresis. The amplified libraries were then single-end sequenced using the Illumina NextSeq. All libraries were loaded at 10 pM. The libraries were combined with 40% PhiX (Illumina). For each run, 3.4 µl of the custom Read 1 primer (3′) was added to the Read 1 primer mix of the NextSeq cartridge (Illumina) to enable sequencing of PhiX and to generate reads starting with the barcoded ISS1 (Table S9) [35]. A custom Index Read primer was also loaded into the NextSeq cartridge according to the manufacturer’s instructions (Table S9) [35].

### TraDIS analysis

For the TraDIS analysis, reference sequences were *de novo* assembled with the *assembleBAC* v2.0 pipeline via the integrated *Shovill* v. 1.1.0 pipeline under the standard parameters (https://github.com/avantonder/assembleBAC). The *bakta* v1.10.3 pipeline was used to annotate all genomes while using the HL_77_2 *S. canis* genome as a reference for all [44]. Raw TraDIS FASTQ files were analysed using the Bio-TraDIS scripts made available by the Sanger Wellcome Trust Institute [45]. The pipeline filtered and removed reads according to the transposon tag CAGAAAACTTTGCAACAGAACC. Each library was mapped to its own reference genome using SMALT, a short read mapper. The default transposon tag mismatch of 0 was maintained, and a mapping threshold of 100% was set (SMALT parameter *y*=1). The TraDIS essentiality pipeline from the Bio-TraDIS scripts was used to generate the essential genomes of all libraries [45]. Insertion plots were generated with Artemis [46].

### Comparative analysis of pyogenic streptococci

Input pools for *S. equi* subsp. *equi*, *S. pyogenes* and *S. agalactiae* were taken from previous studies and analysed through the TraDIS pipeline in the same THB media as *S. canis* [35,47, 48]. The *bakta* v1.10.3 pipeline was used to annotate all genomes while using the HL_77_2 *S. canis* genome as a reference for all [44]. Annotations were standardized with *Panaroo* v. 1.5.2 by grouping identical genes according to group codes generated by the pipeline [49]. Identification of the essential gene KEGG pathways was performed with the annotations generated by *bakta* [44,50]. Plots for the visualization of the essential genes in the glycolytic and peptidoglycan biosynthesis pathways were generated with Inkscape (https://inkscape.org/).

## Results

### The shared essential genome of *S. canis*

**Fig. 2.**
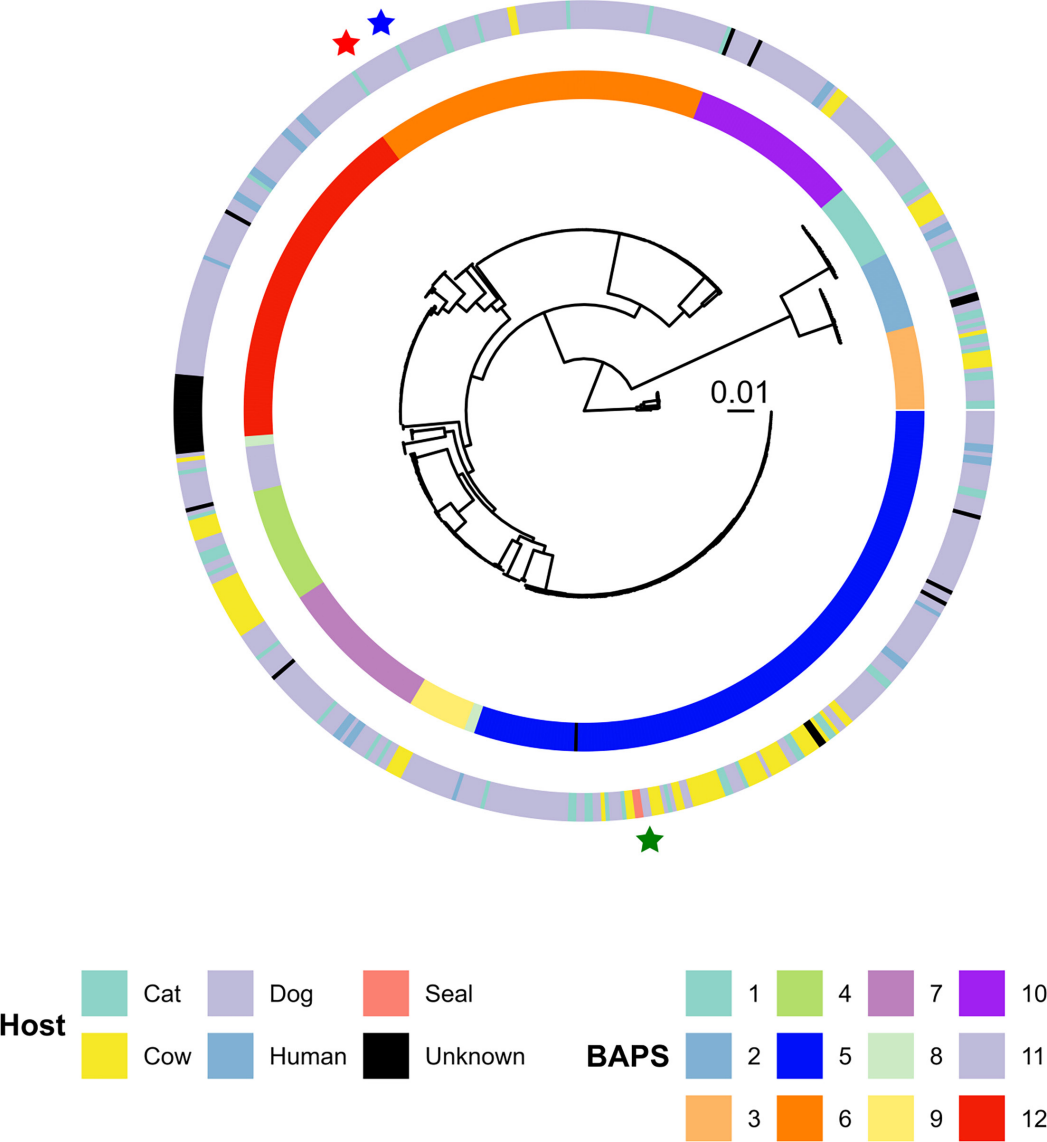
Maximum likelihood SNPs tree of 598 isolates of *S. canis* rooted to *Streptococcus dysgalactiae* and annotated with Host and BAPS cluster. The tree scale represents the number of substitutions per site. Stars represent the location of *S. canis* strains AGF957 (green star), AGF1032 (red) and AGF1037 (blue).

A core genome phylogenetic tree was produced to select three clinically relevant strains for this study. The dataset is composed of 598 public isolates and was partitioned into 12 distinct BAPS clusters with *fastBAPS* (Fig. 2, Table S1). The two largest pathogenic clusters corresponded to BAPS-5, a multi-host cluster consisting of bovine, feline, human, pinniped and canine isolates, and BAPS-6, a clonal cluster composed almost exclusively of ST43 isolates. To assess any potential differences in essentiality for *S. canis* strains harbouring different types of the major virulence factor SCM, we selected two canine isolates from the BAPS-6 cluster representing strains that contain either virulence factor SCM-I implicated in IgG and plasminogen binding or SCM-II implicated in fibrinogen binding (AGF1032 and AGF1037). One canine isolate was taken from the BAPS-5 cluster (AGF957) to represent the essential genomes of the most prominent and pathogenic *S. canis* lineages.

We utilized massive parallel sequencing to generate data from three *S. canis* transposon libraries and for each isolate assessed the number of reads mapping back to the genome of *S. canis*, the number of unique insertions, as well as the library saturation. All three transposon libraries produced more than 7 million mapped reads each. The number of unique mutants for each library ranged from 18,310 mutants for AGF957 to 61,298 mutants for AGF1037. We calculated the saturation of each library (the size of the *S. canis* genome divided by the number of unique insertion sites) and found that transposons had inserted themselves every 32 bp for AGF1037, 55 for AGF957 and 107 for AGF1032 (Table 1). When calculating the total amount of reads divided by the number of unique insertion sites, we found that AGF957 had a read density per unique insertion of 192, AGF1032 had 1,046 and AGF1037 had 283. More than 100 reads were attributed to each potential unique mutant, highlighting the read density of the sequenced library (Table 1). To determine the library coverage for each strain, we evaluated the total amount of genes that had at least one transposon insertion and divided that number by the total amount of genes present in each strain. For AGF957, this led to a library coverage of 91.9% (1,838 genes have at least one insertion), 92.1% for AGF1037 (1,758 genes with at least one insertion) and 86.5% for AGF1032 (1,611 genes with at least one insertion) (Table 1). Genome-wide insertion plots were generated for each library, showing that the transposon had inserted itself in virtually every part of the genome of the individual *S. canis* strains (Figs S1–S3). Finally, we evaluated the validity of the essential gene results using changepoint plots for each library (Fig. S4). Essentiality plots, when the library is saturated, present a bimodal distribution when plotting the frequency of genes in the target genome based on their insertion indices (number of unique transposon insertions relative to the size of the gene) [32]. This allows us to differentiate essential and non-essential genes by using the two peaks as cutoff points where essential genes are found before the second peak and non-essential genes are identified after the second peak. The essential changepoints here were determined to range from 0.0012 for AGF957 to 0.0044 for AGF1037. Ambiguous changepoints indicating genes that were unable to be called as either essential or non-essential were determined to range from 0.0017 for AGF957 to 0.0061 for AGF1037.

**Table 1.** TraDIS statistics for the essential genome analysis of the *S. canis* isolates AGF957, AGF1032 and AGF1037 Library saturation is defined as the size of the *S. canis* genome for each strain divided by the total number of unique insertion sites, giving the average distance between each unique transposon insertion on the genome of *S. canis* in bp. Library coverage is defined as the number of genes with at least one unique insertion divided by the total number of genes in each strain of *S. canis*.

*S. canis* isolate	Reads mapped	Percentage of mapped reads	Unique insertion sites	Library saturation (in bp)	Library coverage (in %)
AGF957 (BAPS-5)	7,278,316	70.01	37,796	55	91.9
AGF1032 (BAPS-6)	19,164,019	62.14	18,310	107	86.5
AGF1037 (BAPS-6)	17,381,593	59.08	61,298	32	92.1

**Fig. 3.**
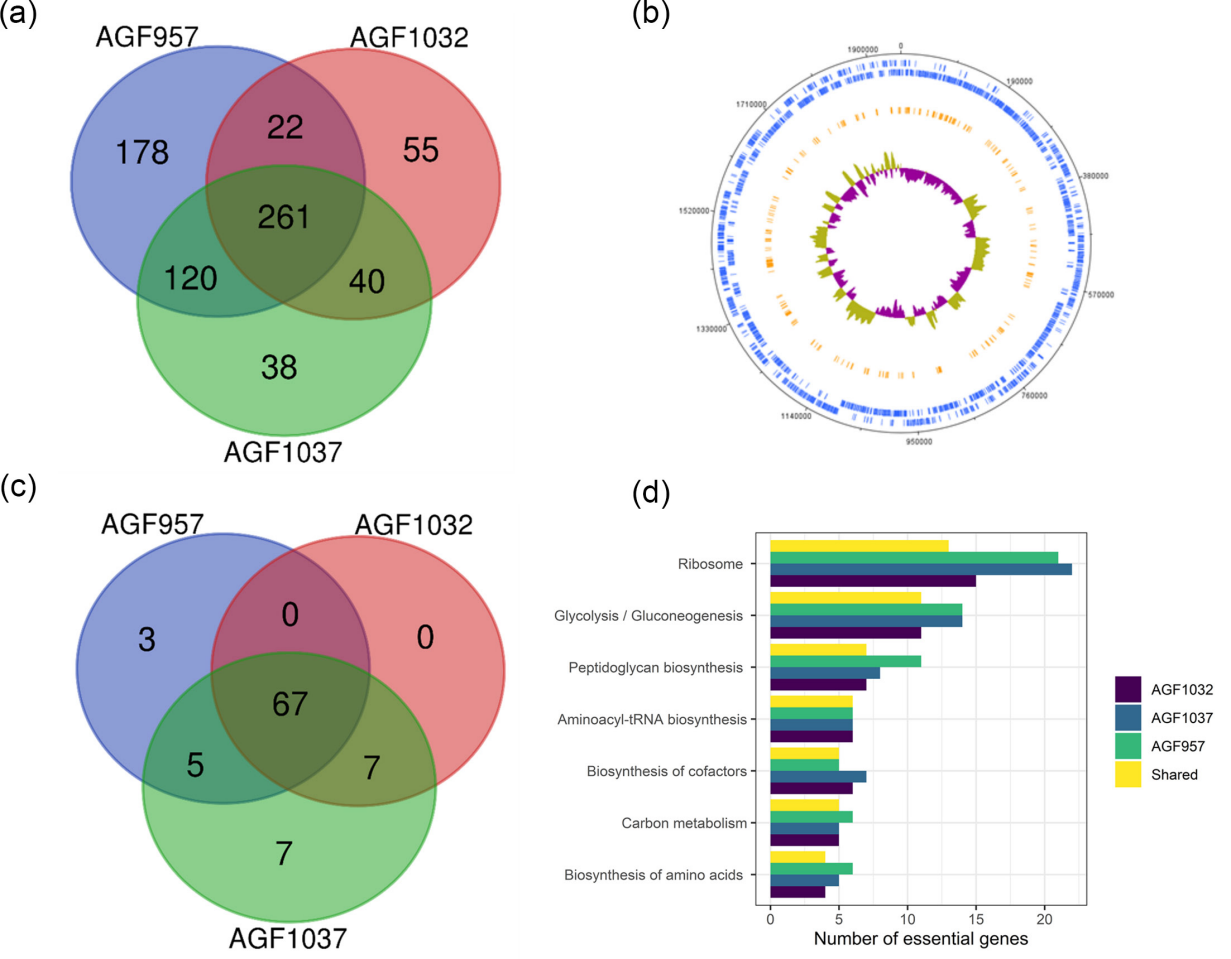
(a) Venn diagram representing the overlapping shared essential genes of S. canis strains AGF1032, AGF1037 and AGF957. (b) DNA plot representing the genomic localization of the shared essential genes of S. canis. (c) Venn diagram representing the overlapping KEGG pathways of the S. canis isolates. (d) Bar plot representing the top shared essential KEGG pathways of S. canis. Blue rings in (b) represent the coding regions on the forward and reverse DNA strands. The orange ring represents the shared essential genes of the three S. canis isolates and the peaks correspond to positive (yellow) and negative (purple) GC skews.

Next, we compared the essential genes between all three isolates to assess the overlap of their essential genomes. When duplicate genes were excluded, we found that AGF957 had the highest number of essential genes, accounting for 580 in total (29% of the total 2,000 genes in this strain) (Table S2). The number of essential genes in AGF1037 reduced significantly, with only 458 (24% of 1,909 total genes). Finally, AGF1032 had the lowest number of essential genes with 377 (20% of 1,863 total genes) (Fig. 3a, Tables S3 and S4). Significant overlap was observed between all three strains, with 260 essential genes being shared between them (Table S5). This set of genes will be defined as the shared essential genome of *S. canis* throughout this study. Of note, we observe that AGF957 had the highest amount of unique essential genes (178 in total) and that AGF1037 had the lowest with 38. AGF1037 and AGF957 further demonstrated the highest amount of overlapping essential genes between two strains, accounting for 120 out of the total 380 genes uniquely shared between them. In contrast, AGF957 and AGF1032 had the least amount of overlapping essential genes, with only 22 uniquely shared genes between them out of the total 283. When plotting the shared essential genes on the genome of AGF1037, we found that the essential genes were distributed across the entire genome of *S. canis* (Fig. 3b).

When analysing the KEGG pathways of *S. canis*, we found 67 pathways which possessed at least one essential gene shared between all three strains of *S. canis* (Fig. 3c). We found seven of these pathways which were shared exclusively between AGF1032 and AGF1037, and five were exclusively shared between AGF1037 and AGF957. We observed that some of these pathways were exclusive to specific strains. We detected that AGF1037 had the largest amount of unique KEGG pathways containing essential genes with seven. AGF957 possessed three unique KEGG pathways, and AGF1032 was the only strain to have none. The unique KEGG pathways that were shared and the KEGG pathways exclusive to a given strain are described in Table 2.

**Table 2.** Table describing uniquely shared essential KEGG pathways and pathways exclusively essential for a given strain

Compared essential genomes	*KEGG pathways*
AGF1032/AGF1037	*KO00785: Lipoic acid metabolism*
	*KO01210 : 2-Oxocarboxylic acid metabolism*
	*KO00020: Citrate cycle (TCA cycle*)
	*KO00310: Lysine degradation*
	*KO01120: Microbial metabolism in diverse environments*
	*KO00380: Tryptophan metabolism*
	*KO00260: Glycine, serine and threonine metabolism*
AGF1037/AGF957	*KO02040: Flagellar assembly*
	*KO00564: Glycerophospholipid metabolism*
	*KO00523: Polyketide sugar unit biosynthesis*
	*KO00521: Streptomycin biosynthesis*
	*KO00300: Lysine biosynthesis*
AGF1037 exclusive	*KO00760: Nicotinate and nicotinamide metabolism*
	*KO04068: FoxO signalling pathway*
	*KO05417: Lipid and atherosclerosis*
	*KO05016: Huntington disease*
	*KO04010: MAPK signalling pathway – fly*
	*KO05208: Chemical carcinogenesis – reactive oxygen species*
	*KO04211: Longevity regulating pathway*
AGF957 exclusive	*KO05111: Biofilm formation – Vibrio cholerae*
	*KO00983: Drug metabolism – other enzymes*
	*KO02026: Biofilm formation – Escherichia coli*

We investigated the KEGG pathways with the most genes declared as essential for all three isolates. We found that ribosomes were by far the top KEGG essential pathway. A total of 13 ribosomal genes were shared between the different isolates. AGF957 and AGF1037 had the highest number of essential genes in this category with 21, followed by AGF1032 with 15 (Fig. 3d). This was then followed by genes falling under the glycolysis/gluconeogenesis pathway with 11 shared essential genes and on average 13 essential genes per isolate. The peptidoglycan biosynthesis pathway was the next major pathway with seven shared genes and, on average, nine essential genes per isolate.

### The unique essential genes of *S. canis*

**Fig. 4.**
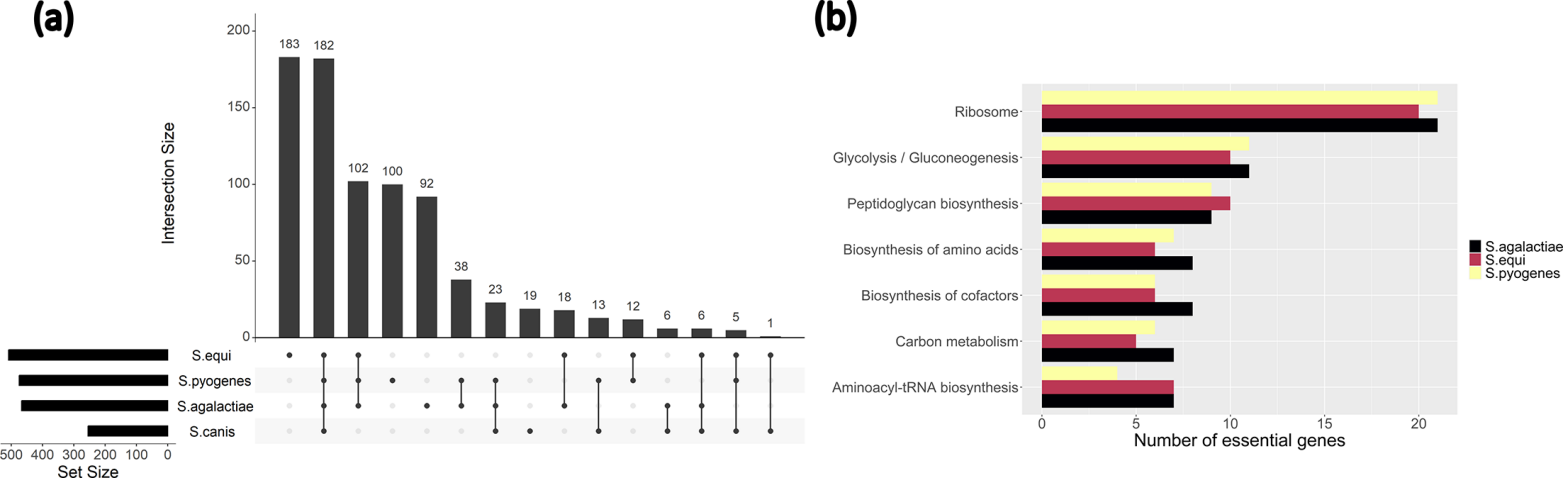
Upset plot representing the shared essential genes of *S. canis* compared to other streptococcal species. Set size represents the number of essential genes for each individual streptococcal species. (a) Intersection size represents the number of shared genes for all possible comparisons of the different streptococcal species. The linked dots below the intersection size bars represent the species that are being compared for gene essentiality. Shared genes can be viewed in Table S5. (b) Bar plot representing the top shared essential KEGG pathways of the different streptococcal species.

We used input pools of previous *Streptococcus* studies conducted with the TraDIS method in THB media to make a comparative analysis with the shared essential genome of *S. canis* in the same conditions. This included TraDIS assays of *S. equi* subsp. *equi* strain Se4047 (a virulent strain that caused strangles in a pony) [35], *S.* agalactiae strain 874391 (a strain part of the hypervirulent and invasive serotype III sequence type 17 lineage in humans and animals) [34] and *S. pyogenes* strain MGAS2221 (belonging to the clinically relevant M1 lineage) [47]. Like our libraries, these strains had input pools that were grown in THB at 37 °C either overnight or to an OD of 0.3 at 600 nm [30,47, 51]. We found that 185 genes in total are shared between all the pyogenic streptococci (Fig. 4a). This shows that 71.1% of the shared essential genome of *S. canis* is commonly shared with the other major pyogenic streptococci. We found a total of 239 genes (91.9%) in the shared essential genome of *S. canis* that were present in either one or more of the other streptococci. *S. agalactiae*, *S. equi* subsp. *equi* and *S. pyogenes*, respectively, had 563, 602 and 544 essential genes (Tables S6–S8). *S. equi* subsp. *equi* had the largest unique essential genome in this comparison with 183 genes exclusive to it. This was followed by *S. pyogenes* with a significantly reduced 100 genes. Excluding the shared essential genome of *S. canis*, *S. agalactiae* produced the smallest unique essential genome with 92 genes. *S. pyogenes* and *S. agalactiae* showed the most essential gene overlap, with 345 total shared and 38 genes shared uniquely between them.

For this comparison, we observed that the most prolific essential KEGG pathway was similar to the *S. canis* essential genome, with the ribosome pathway containing 21 genes detected as essential in *S. pyogenes* and *S. agalactiae* and 20 genes in *S. equi* subsp. *equi* (Fig. 4b). The glycolysis pathway of *S. agalactiae* and *S. pyogenes* was the next major pathway represented with 11 essential genes, whereas *S. equi* subsp. *equi* only contributed 10 genes. The peptidoglycan biosynthesis pathway was represented with nine essential genes of *S. pyogenes* and *S. agalactiae* and with ten genes of *S. equi* subsp. *equi*.

With this analysis, we identified 19 essential genes unique to the *S. canis* species in the shared essential genome when compared to the other major pyogenic streptococci. Nine of these genes corresponded to gene variants called essential in the other streptococci. According to these assignments, only four genes can be defined as uniquely essential in *S. canis*: a phosphate/phosphonate ABC transporter ATP-binding protein, a GntR family transcriptional regulator, the *ldh* gene coding for l-lactate dehydrogenase and the *pta* gene coding for phosphate acetyltransferase.

**Fig. 5.**
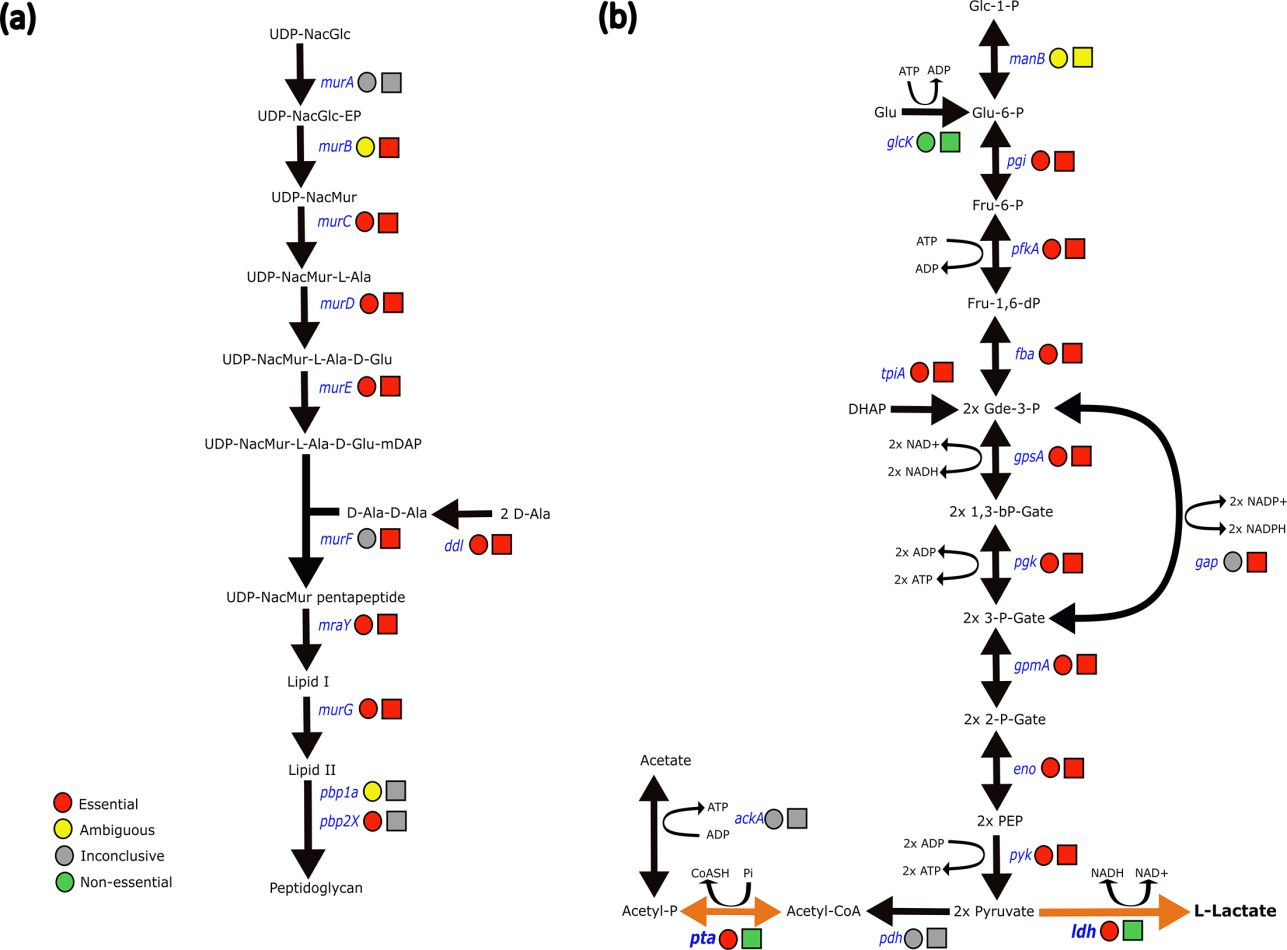
Graphical representations of (a) the peptidoglycan biosynthesis pathway and (b) the glycolysis pathway associated with gene essentiality. Metabolites are represented as dark text; enzymes are represented as blue text. Circles represent gene essentiality in *S. canis*, squares represent gene essentiality in all other streptococci. Reactions are represented by arrows. Reactions that are uniquely essential to *S. canis* are represented by orange arrows.

To further evaluate the gene essentiality of *S. canis* compared to the other three streptococcal species, we decided to investigate the pathways in more detail, starting with one of the main pathways, the peptidoglycan biosynthesis pathway. Sequence comparison revealed that 7 of the 11 reaction steps of the peptidoglycan biosynthesis pathway were essential to all strains of *S. canis* whereas the other streptococcal species had 8 (Fig. 5a). Our data demonstrate that the essentiality of the *murA* gene was inconclusive for *S. canis* (essential in AGF957 and IMT49926, non-essential in AGF1032 and AGF1037) and the other streptococcal species (essential in *S. pyogenes*, non-essential in *S. agalactiae* and *S. equi*). We declared that *murB* was ambiguous in *S. canis* due to it being called essential in all strains except AGF1032 where it was called ambiguous. The other streptococcal species showed that *murB* was essential. The penicillin-binding proteins were declared as inconclusive for the other streptococcal species. In contrast, we found that *pbp1a* was ambiguous in *S. canis* (essential in all strains except AGF957, where it was called ambiguous) and that *pbp2X* was essential.

Subsequently, we investigated the individual essential genes in the glycolytic pathway and the downstream acetate and lactate metabolic pathways (Fig. 5b). We found that 9 out of the 16 reaction steps in glycolysis were essential for both *S. canis* and the other streptococci. In this pathway, only two genes were exclusively essential to *S. canis*: the *ldh* gene, which generates the protein catalysing the reaction of pyruvate to l-lactate, and the *pta* gene, producing the protein catalysing the reaction of acetyl-CoA to acetyl phosphate. To ensure that essentiality was clearly distinguishable between streptococcal species, we compared the insertion zones of *ldh* and *pta* in *S. canis* strain AGF1037 to its closest relative in the dataset: *S. pyogenes.* Both genes had a higher number of insertion sites in *S. pyogenes* as compared to *S. canis*, which possessed almost none (Figs S5 and S6). This supports the difference in essentiality called by the Bio-TraDIS pipeline between the different species.

Within this comparison, we describe the unique essential genes of *S. canis*, which include key genes in the lactate metabolism and acetate metabolism pathways. These genes could one day serve as targets for selective treatments against *S. canis*.

## Discussion

By using TraDIS on three distinct clinical isolates of *S. canis*, we produced the shared essential genome of the pathogen, composed of 260 essential genes. The most overrepresented essential pathways that we detected in *S. canis* were for ribosomal-related genes, peptidoglycan biosynthesis and glycolysis. We found that 91.9% of the shared essential genome of *S. canis* is concordant with the essential genomes of *S. agalactiae*, *S. pyogenes* and *S. equi* subsp. *equi* and that *ldh* and *pta* were uniquely essential to *S. canis*.

As expected, *S. canis* had significant overlap in its shared essential genome when compared to the other pyogenic streptococci in our analysis, similar to what has been described previously with *S. agalactiae*, *S. pyogenes* and *S. equi* subsp. *equi* [34,35]. A study on *S. pyogenes* showed that this similarity could even extend to streptococci outside of the pyogenic species group [33]. The *S. pyogenes* core essential genome was compared to that of *Streptococcus sanguinis* and *Streptococcus pneumoniae*, showing that a total of 120 of the 177 *S*. *pyogenes* core essential genes were shared with them [33]. We provide evidence that this also applies to *S. canis* within the pyogenic species group and, given the significant overlap of the essential genome with *S. pyogenes*, could extend to other species such as *S. sanguinis* and *S. pneumoniae*. Genes that are universally essential in the *Streptococcus* genus could be used as new targets for the development of antimicrobial molecules.

However, we think that the true strength of the TraDIS method is not in its capacity to find similarities but in its ability to delineate differences between pathogens of interest. In our study, we found the unique essential genes *ldh* and *pta*, which is a step towards understanding the unique mechanisms that underlie *S. canis* growth. It has been experimentally shown that deficiencies in l-lactate dehydrogenase encoded by *ldh* are lethal to the oral pathogen *Streptococcus mutans* [51,52]. This is apparently only the case in high glucose environments, which lead the authors to believe that an excess of pyruvate is toxic to *S. mutans* [52]. In the context of infection, blood glucose levels are increased to supplement immune activity against a pathogen [53]. *S. mutans* may degrade the glucose in the host environment as a source of nutrition which would then garner the need for *ldh* to avoid the overaccumulation of pyruvate. A similar phenomenon could be taking place in *S. canis* which would explain the essentiality of the *ldh* gene. The *pta* gene encoding phosphate acetyltransferase is involved in acetate metabolism and has been shown to impact ATP production, stress tolerance and biofilm production in *S. mutans* [54]. However, to our knowledge, there has not been a description of *pta* as an essential gene yet. The *pta* gene is responsible for converting acetyl-CoA into acetyl phosphate, which is then converted into acetate by acetate kinase encoded by *ackA*. A possible reason for *pta* being determined essential could be due to the overaccumulation of acetyl-CoA in the absence of the gene. Streptococci are not able to use the tricarboxylic acid pathway and therefore cannot use acetyl-CoA to start the cycle [55]. If *S. canis* has no option to convert acetyl-CoA into acetyl phosphate, then it is forced to convert acetyl-CoA to either acetoacetyl-CoA feeding into the fatty acid biosynthesis pathway or into acetaldehyde to then produce ethanol [55]. The increased production of ethanol could become lethal to *S. canis*, which could explain the essential status of *pta*.

In this study, we confirm the hypothesis that the essential genome of *S. canis* has significant overlap with the other pyogenic streptococci in our comparison and that *S. canis* has its own unique essential genes. These findings have implications for the generalized treatment of pyogenic streptococci where any new antimicrobials that are found to act against *S. pyogenes*, *S. agalactiae* and *S. equi* subsp. *equi* could also be used against *S. canis*. We also see the potential for more targeted treatments for *S. canis* with *ldh* and *pta* being possible candidates to avoid dysbiosis in the host environment. The main limitations of this study are mainly the lack of experimental validation for the impact of *ldh* and *pta* deletions in *S. canis*. Future studies could test the impact of *ldh* and *pta* by using CRISPRi-mediated knockdowns as has been previously conducted on *S. pneumoniae* [56]. Finally, as this is a study centred on *S. canis*, we did not make any assertions concerning the potential unique essential genes of *S. agalactiae*, *S. pyogenes* and *S. equi* subsp. *equi*. As openly available TIS libraries increase in abundance, we have a greater opportunity to consolidate the results of different studies to create a meta-analysis of not just the essential genome of relevant pathogens but also their fitness in similar contexts of infection. Studies combining multi-omics approaches have started to emerge for a more refined analysis of the pathogenesis of, for example, sepsis-causing bacteria [57]. The combination of TraDIS and RNA-seq has already been conducted on *S. pyogenes*, identifying new pathogenic mechanisms for it in the context of necrotizing myositis [58]. A multi-omics approach could therefore further elucidate the pathogenesis of *S. canis* and clinical pathogens as a whole.

In summary, our sequence analyses demonstrated that 90.4% of the shared essential genome of *S. canis* is concordant with the essential genomes of *S. agalactiae*, *S. pyogenes* and *S. equi* subsp. *equi*. This finding supports the translatability of finding treatments against one pyogenic *Streptococcus* versus another. The remaining genes represent the unique essential genome of *S. canis* which offers two genes which could serve as specific targets for *S. canis* treatment. These include the *ldh* gene involved in lactate metabolism and the *pta* gene involved in acetate metabolism.

## Supplementary material

10.1099/mgen.0.001701 Uncited Supplementary Material 1.

10.1099/mgen.0.001701 Uncited Supplementary Material 2.

## Abbreviations

BAPS: Bayesian analysis of the population structure
BHI: brain heart infusion
HITS: high-throughput insertion tracking by deep sequencing
INSeq: insertion sequencing
THB: Todd Hewitt Broth
TIS: transposon-insertion sequencing
Tn-Seq: transposon sequencing
TraDIS: transposon-directed insertion-site sequencing
