# Supplementary material for: Unravelling the unique essential genes of Streptococcus canis through transposon-directed insertion-site sequencing
Source: Microb Genom. 2026 May 12;12(5):001701. doi: 10.1099/mgen.0.001701 (PMC13167042; doi:10.1099/mgen.0.001701)
Supplement: Uncited Supplementary Material 1. [file mgen-12-01701-s001.pdf]

a

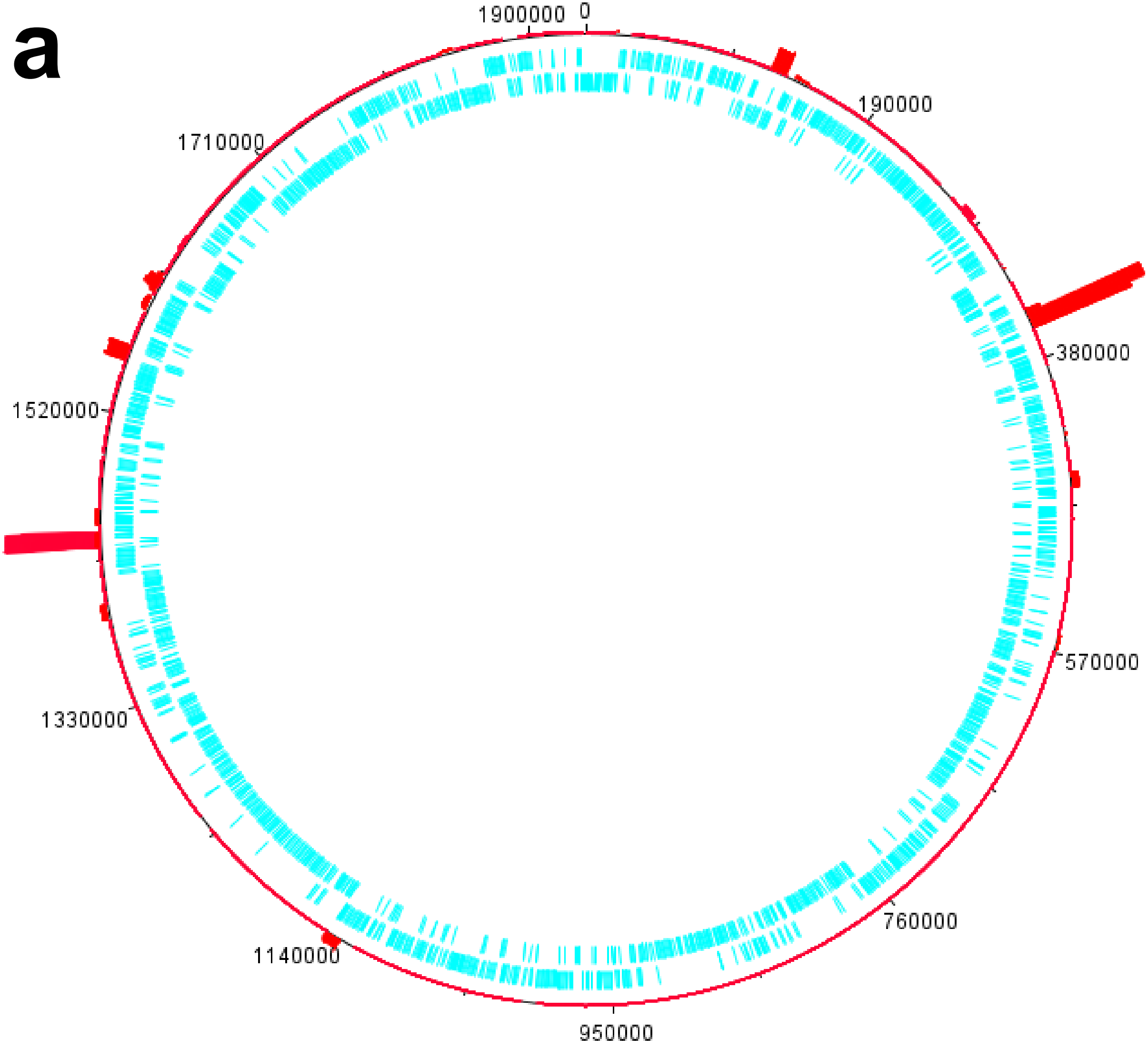
0
190000
380000
570000
760000
950000
1140000
1330000
1520000
1710000
1900000

b

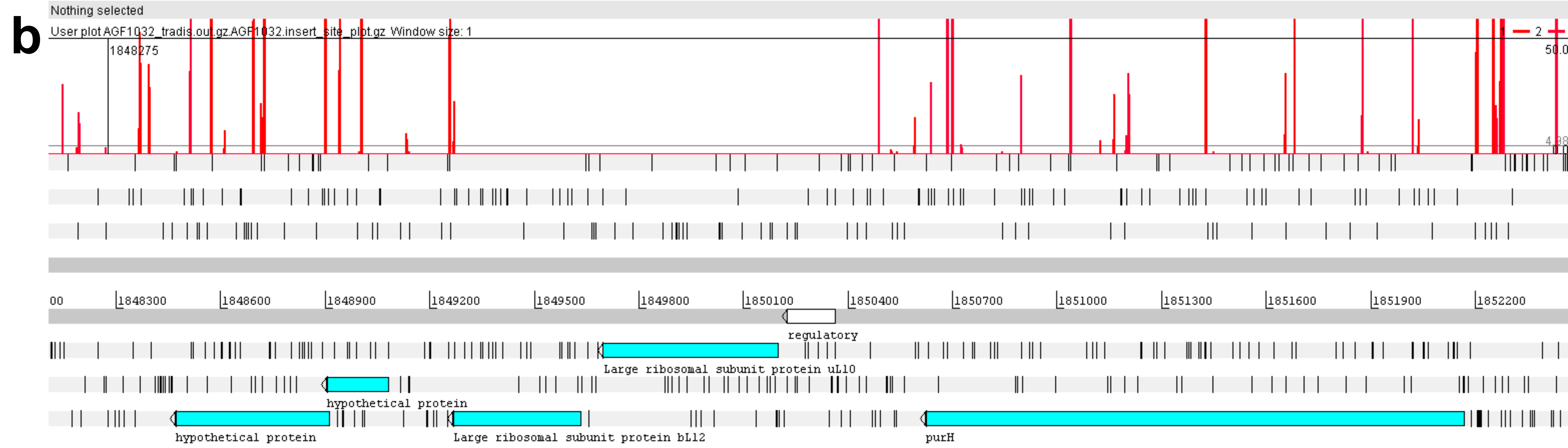

Fig. S1: Insertion plot of AG1032 (a) and an example of essential genes in AGF1032 (b). Red bars in both figures represent insertion zones of the ISS1 transposon. Blue bars represent DNA coding regions. Ribosomal genes were used to emphasise the insertion differences between essential and neighbouring non-essential genes.

a

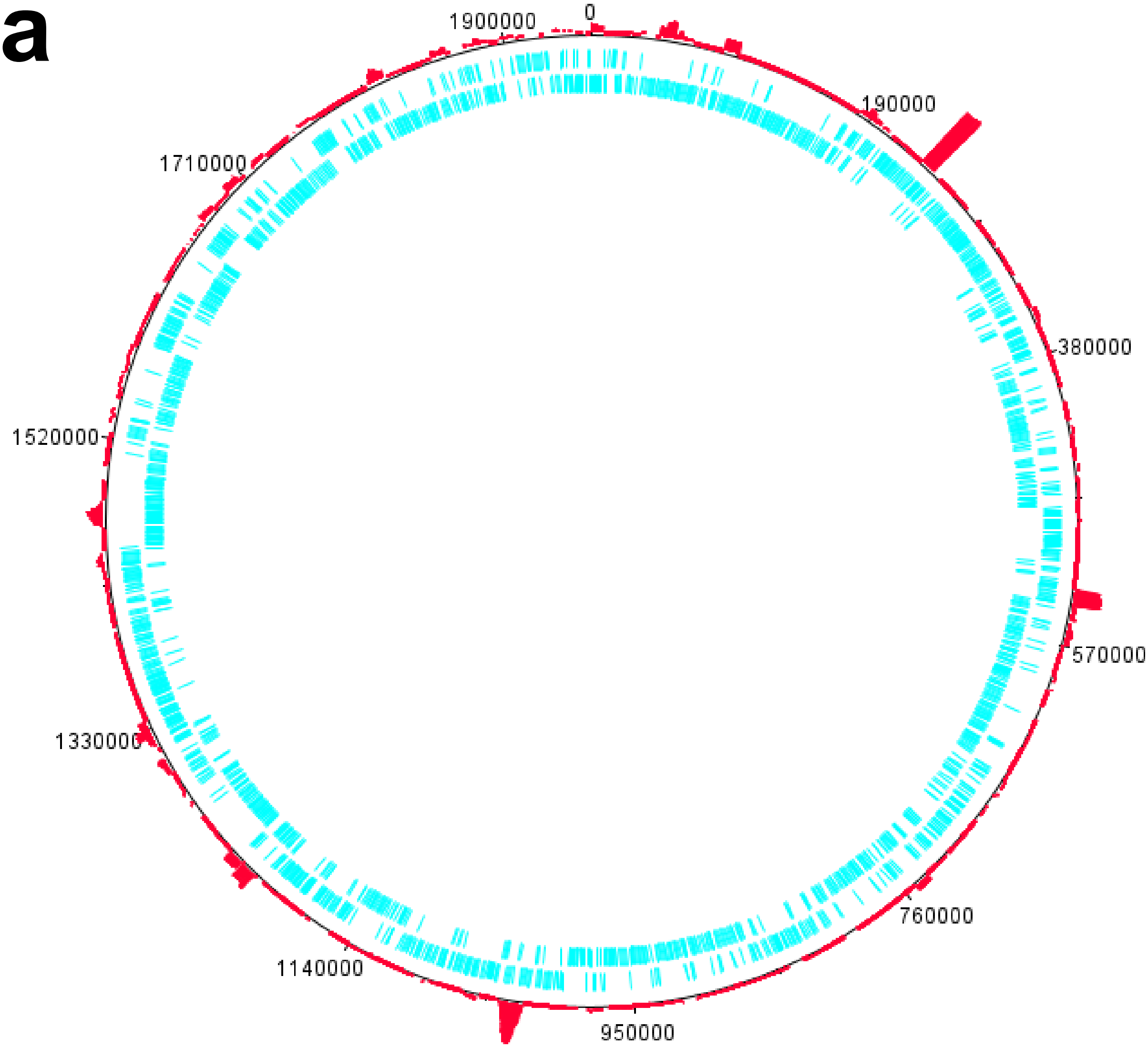

0
190000
380000
570000
760000
950000
1140000
1330000
1520000
1710000
1900000

b

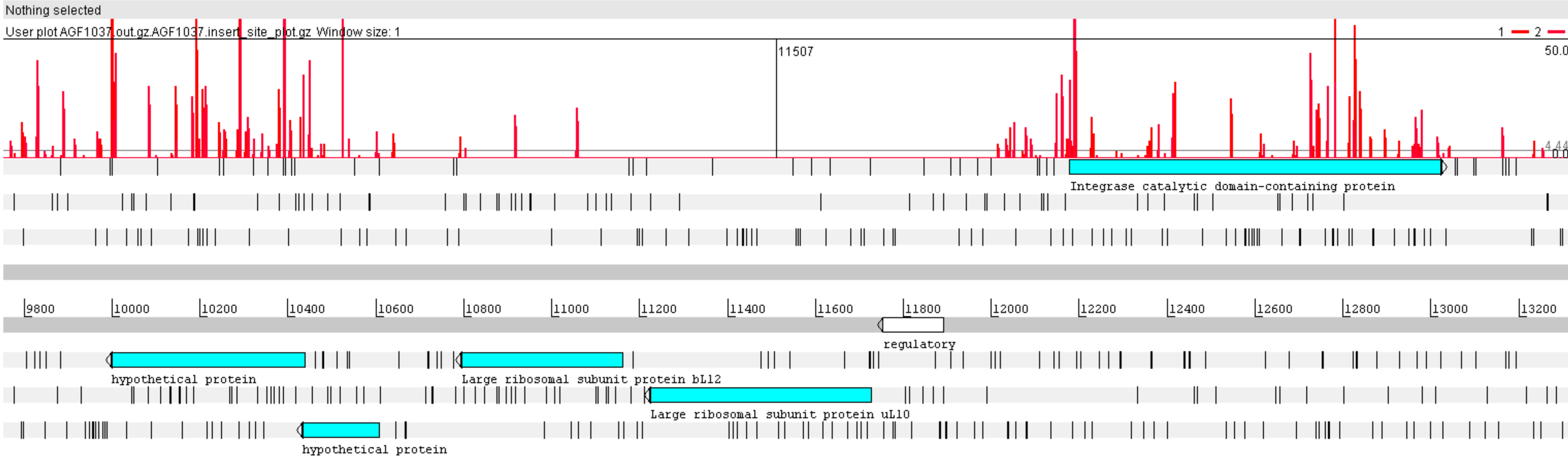

Fig. S2: Insertion plot of AG1037 (a) and an example of essential genes in AGF1037 (b). Red bars in both figures represent insertion zones of the ISS1 transposon. Blue bars represent DNA coding regions. Ribosomal genes were used to emphasise the insertion differences between essential and neighbouring non-essential genes.

a

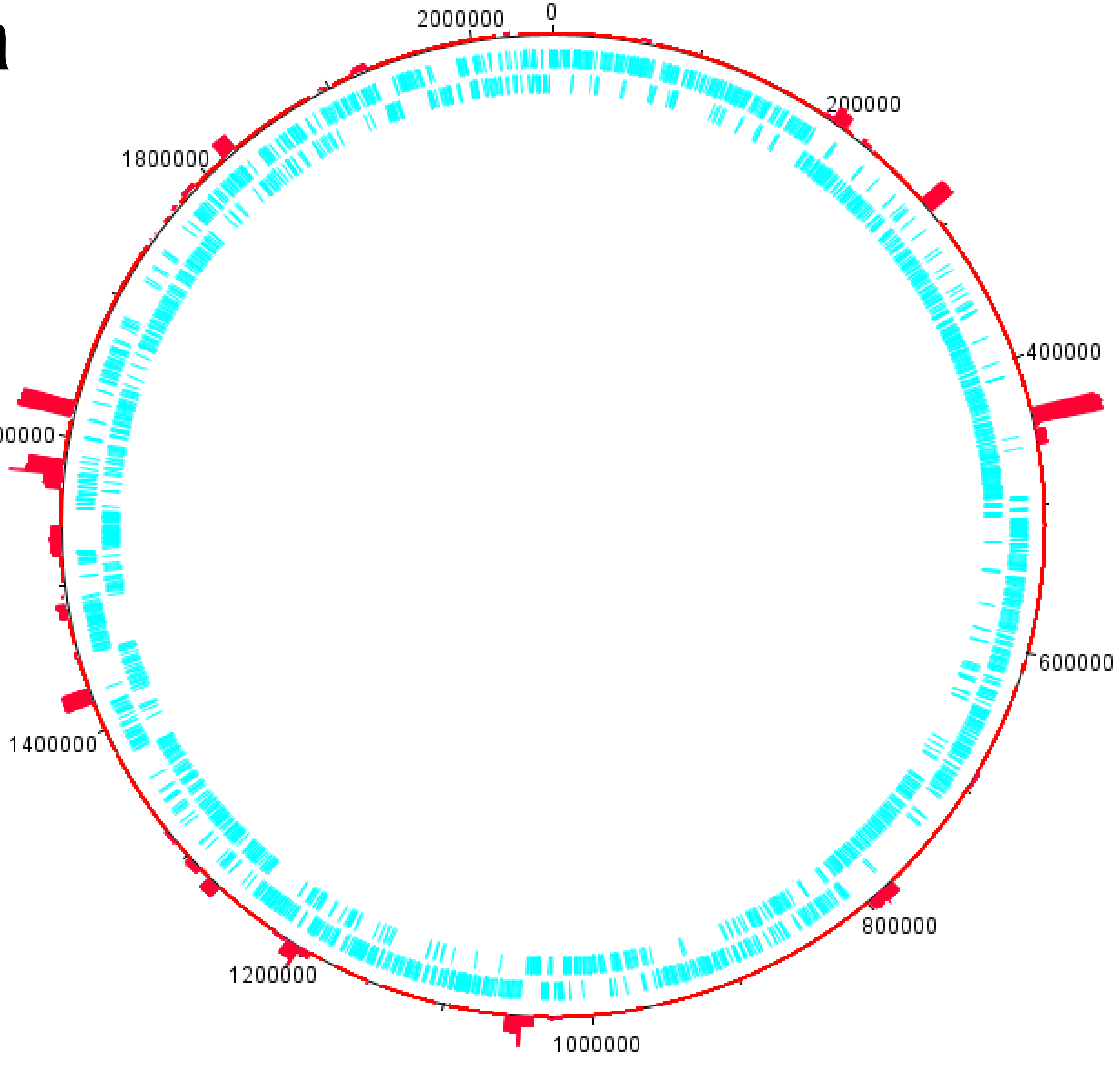

0
200000
400000
600000
800000
1000000
1200000
1400000
1600000
1800000
2000000

b

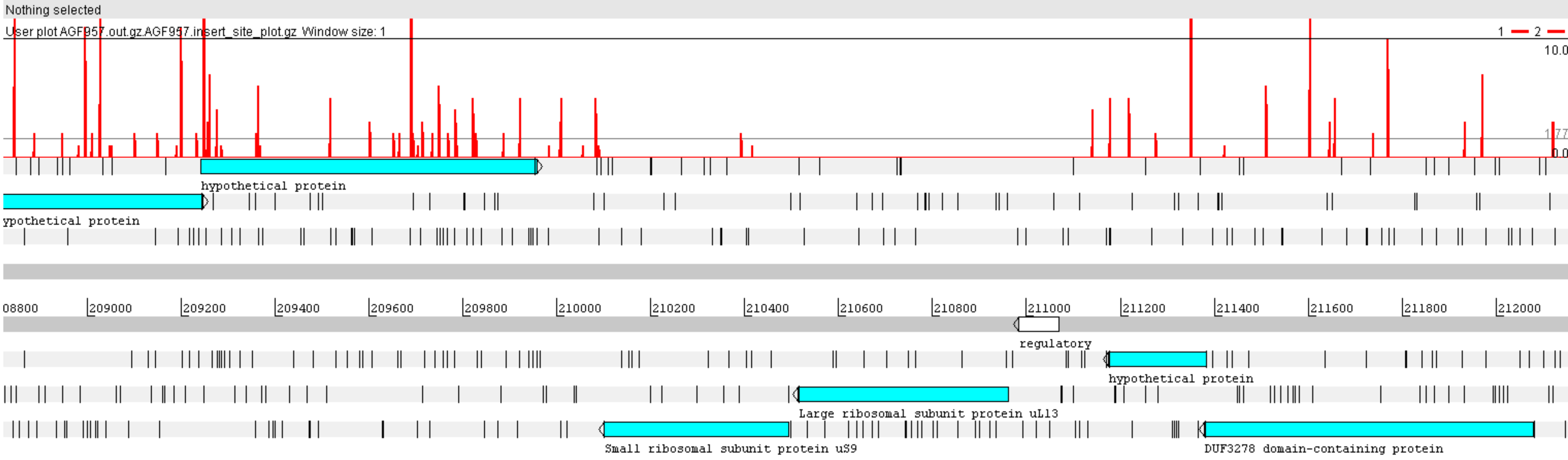

Fig. S3: Insertion plot of AG957 (a) and an example of essential genes in AGF957 (b). Red bars in both figures represent insertion zones of the ISS1 transposon. Blue bars represent DNA coding regions. Ribosomal genes were used to emphasise the insertion differences between essential and neighbouring non-essential genes.

a

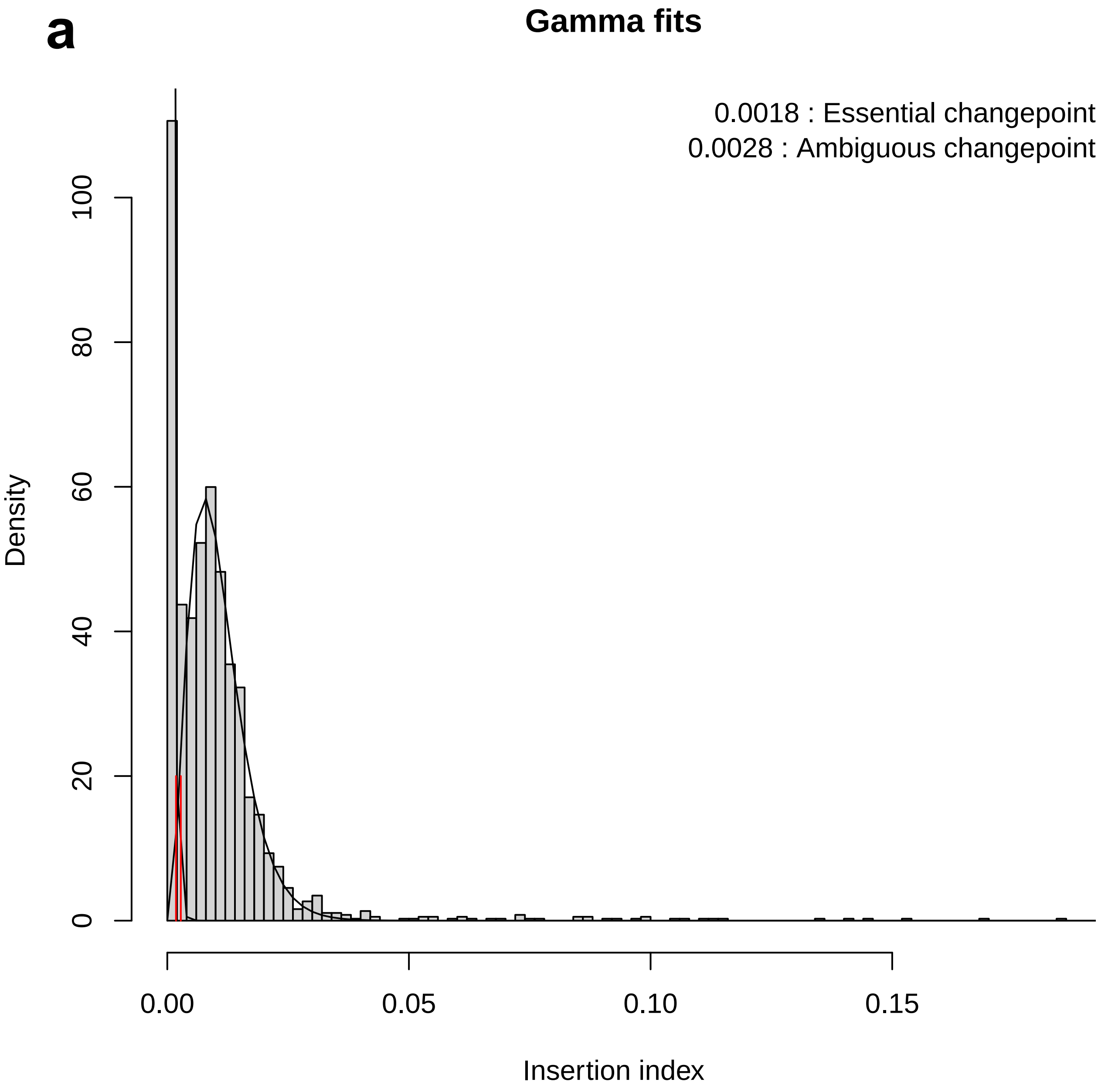

Gamma fits
0.0018 : Essential changepoint
0.0028 : Ambiguous changepoint
Density
0
20
40
60
80
100
0.00
0.05
0.10
0.15
Insertion index

b

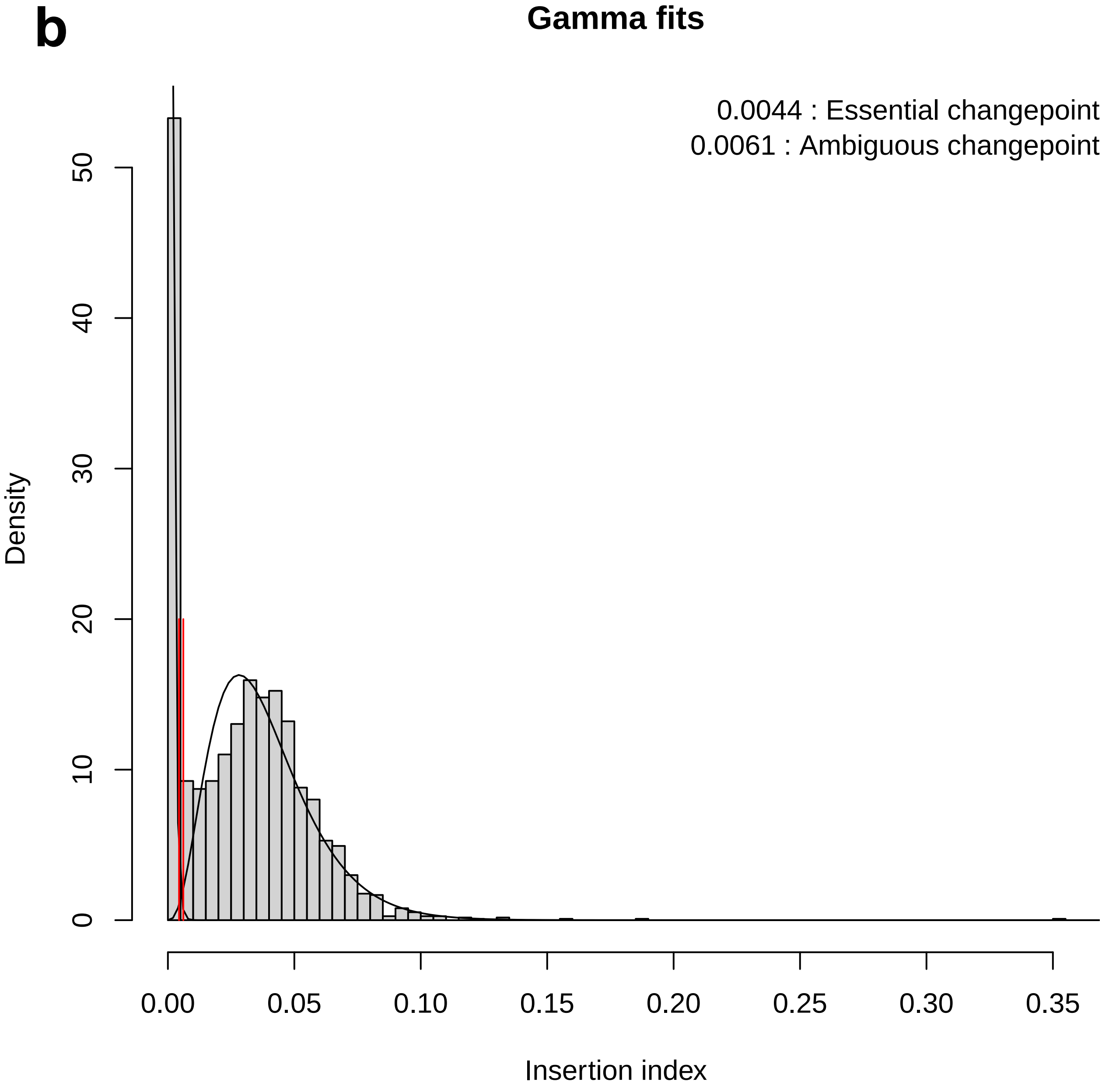
Gamma fits
0.0044 : Essential changepoint
0.0061 : Ambiguous changepoint
Density
0
10
20
30
40
50
0.00
0.05
0.10
0.15
0.20
0.25
0.30
0.35
Insertion index

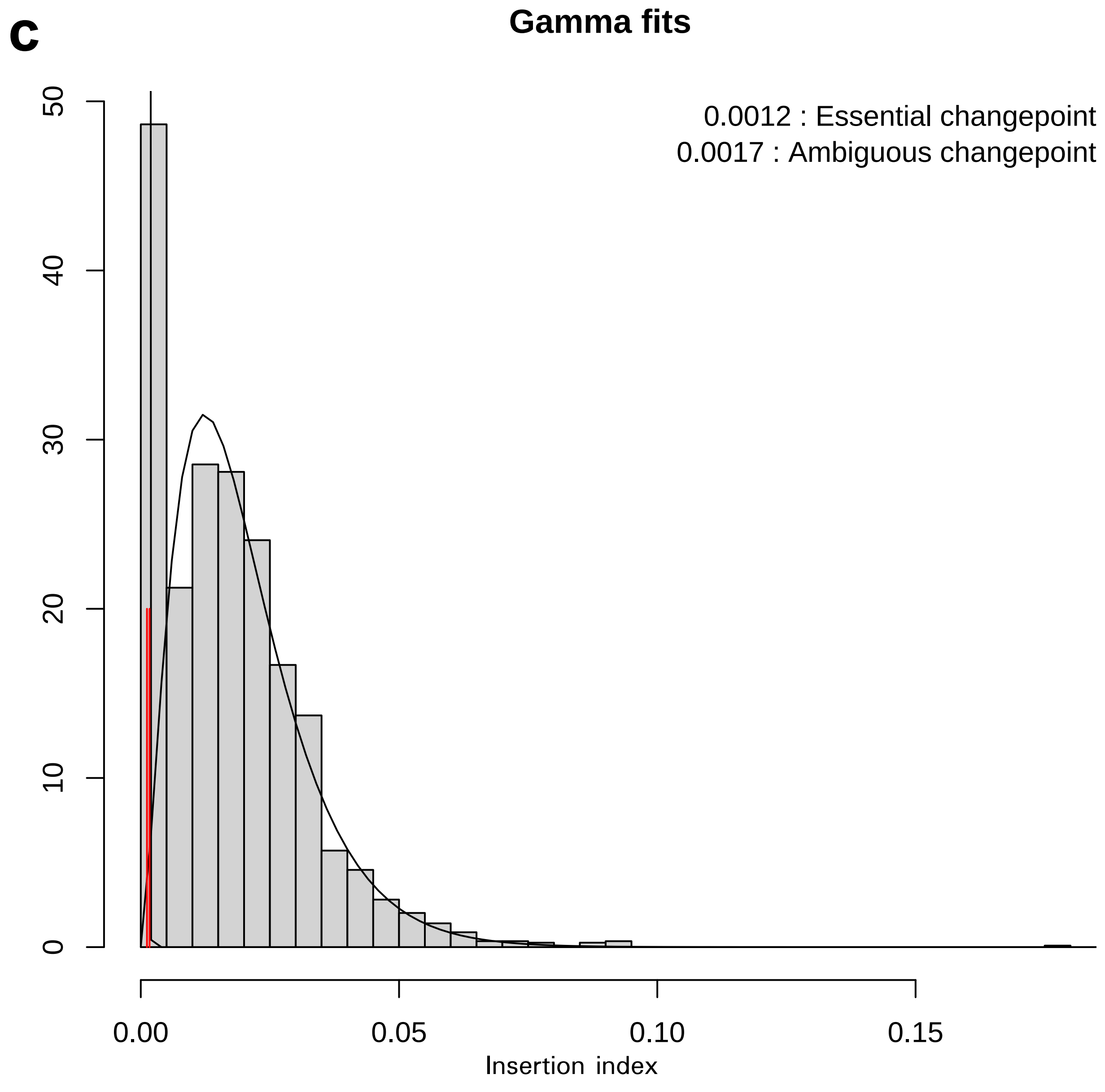

Fig. S4: Essential changepoint plots of AGF1032 (a), AGF1037 (b), and AGF957 (c).

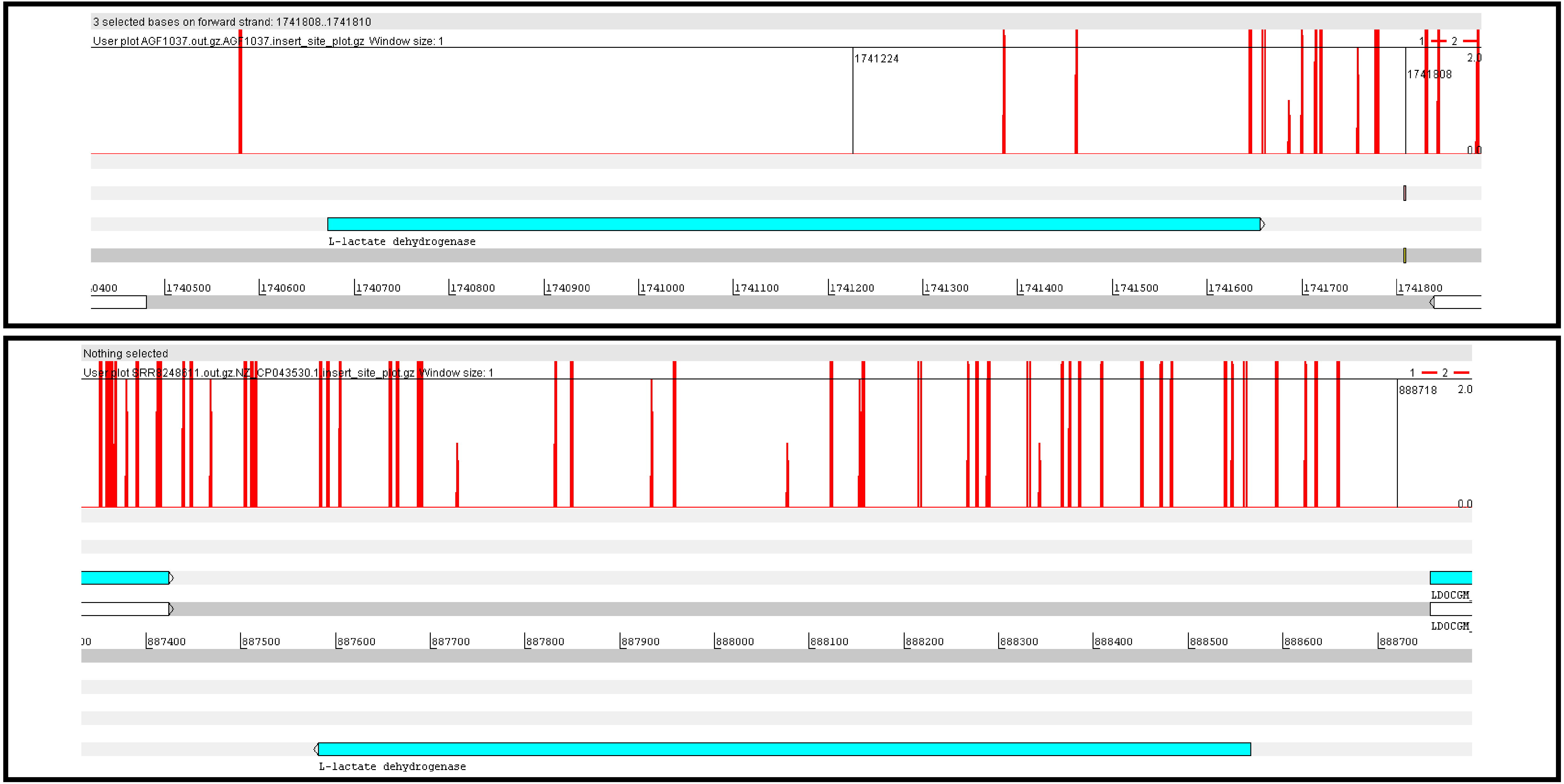

Fig. S5: Transposon insertion sites within L-lactate dehydrogenase in *S. canis* (top plot) and *S. pyogenes* (bottom plot). Red bars indicate insertion sites with heights proportional to the number of reads mapping to them.

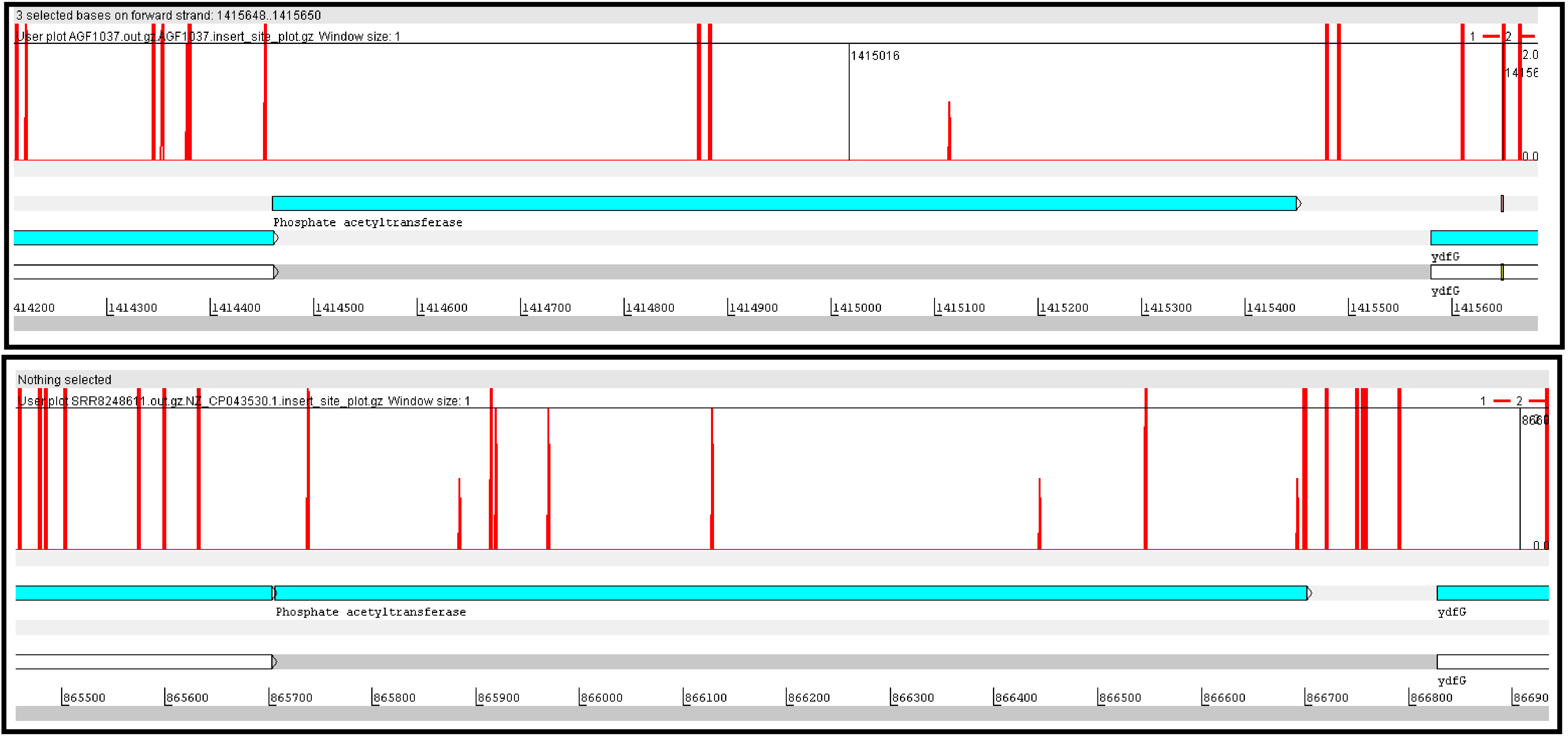

Fig. S6: Transposon insertion sites within Phosphate acetyltransferase in *S. canis* (top plot) and *S. pyogenes* (bottom plot). Red bars indicate insertion sites with heights proportional to the number of reads mapping to them.
